# Reducing stigma toward mental illness and substance use issues in primary health care in Chile: Protocol of a cluster controlled trial study

**DOI:** 10.3389/fpsyt.2022.1083042

**Published:** 2022-12-20

**Authors:** Jaime C. Sapag, Carolina Traub, Paola R. Velasco, Tamara Arratia, Rubén Alvarado, Marcela Aracena, Fernando C. Poblete, Luis Villarroel, Paulina Bravo, Cinthia Álvarez-Huenchulaf, Ana Jofré Escalona, Nelson Vargas-Malebrán, Sireesha Bobbili, Inés Bustamante, Akwatu Khenti, Patrick W. Corrigan

**Affiliations:** ^1^Departamento de Salud Pública, División de Salud Pública y Medicina Familiar, Escuela de Medicina, Facultad de Medicina, Pontificia Universidad Católica de Chile, Santiago, Chile; ^2^Departamento de Medicina Familiar, División de Salud Pública y Medicina Familiar, Escuela de Medicina, Facultad de Medicina, Pontificia Universidad Católica de Chile, Santiago, Chile; ^3^Dalla Lana School of Public Health, University of Toronto, Toronto, ON, Canada; ^4^Centre for Addiction and Mental Health, Toronto, ON, Canada; ^5^Departamento de Salud Pública, Escuela de Medicina, Facultad de Medicina, Universidad de Valparaíso, Valparaíso, Chile; ^6^Programa de Salud Mental, Escuela de Salud Pública, Facultad de Medicina, Universidad de Chile, Santiago, Chile; ^7^Escuela de Psicología, Facultad de Ciencias Sociales, Pontificia Universidad Católica de Chile, Santiago, Chile; ^8^Escuela de Enfermería, Facultad de Medicina, Pontificia Universidad Católica de Chile, Santiago, Chile; ^9^Facultad de Salud Pública y Administración, Universidad Peruana Cayetano Heredia, Lima, Peru; ^10^Illinois Institute of Technology, Chicago, IL, United States

**Keywords:** stigma, mental illness and substance use issues, protocol, controlled trial study, primary care, healthcare workers, contact-based intervention, implementation science

## Abstract

**Background:**

Chile is implementing a Community Mental Health Model with a strong role of primary health care (PHC). PHC has great potential to early detection and provision of accessible and coordinated services to people who present mental illness and/or substance use issues (MISUI). However, stigma toward people with MISUI among PHC professionals is a significant barrier to accessing good quality of care. A wealth of literature supports the importance of reducing stigma for this population. The main goal of this research project is to determine the effectiveness of a comprehensive anti-stigma intervention in reducing stigmatizing attitudes and behaviors among PHC providers toward individuals with MISUI in the Chilean context, using Centros de Salud Familiar (CESFAMs) as the point of intervention.

**Methods:**

The intervention is based on an initiative that was previously developed in Canada and then also pilot-tested in Lima, Peru, with the Center for Addiction and Mental Health (Ontario, Canada). The model will be culturally adapted with CESFAM PHC provider and user inputs to be relevant and valid to Chile. The 18-month intervention includes five (5) components that are simultaneously implemented in CESFAMs: (1) Develop a Team of Local Champions in each intervention CESFAM, comprising PHC providers and users; (2) Analysis of Internal CESFAM Policies, Procedures, and Protocols to determine areas of improvement in service delivery for individuals with MISUI; (3) Raising Awareness of stigma toward MISUI using various forms of media within the CESFAM; (4) Innovative Contact-Based Education workshops on anti-stigma and recovery principles, co-lead by academic/clinical trainers and a person with lived experience of MISUI; and (5) Recovery-Based Arts, a multi-week arts workshop for PHC providers and users to produce artwork related to MISUI and recovery, culminating in an exhibition to showcase artwork for the CESFAM providers, users, and community. The expected intervention outcomes are the following: Participation in the experimental group will result in a significant decrease in stigmatizing attitudes among PHC providers toward individuals with MISUI compared with the control group as measured by the Chilean version of the Opening Minds Scale for Health Care Providers Scale (OMS-HC); Participation in the experimental group will result in a significant decrease of PHC users experiences of stigma conveyed by PHC providers compared with the control group as measured by the Internalized Stigma of Mental Illness (ISMI) scale, validated for the Chilean population. The changes in attitudes and behaviors within the experimental group will be sustained over time as measured at 6 months-follow-up. To evaluate the effectiveness of this 18-month intervention, a 4-year, two-arm, cluster-randomized controlled trial is proposed, with CESFAMs being the unit of randomization (or “cluster”). Implementation Science approach will be taken to measure relevant implementation outcomes for each component of the intervention, and through qualitative data collection with CESFAM providers and authorities. Data analysis will be carried out using SAS 9.4 (specifically, using POC MIXED and PROC GENMOD) and R 3.5. Mixed-effect modeling will used for both PHC provider and user data, which will include individuals and CESFAMs as random effects and group (intervention/control) as fixed effects.

**Discussion:**

This study represents a new stage of relevant and innovative research in mental health and stigma in Chile that will contribute to improving access and quality of care for people with MISUI. Evaluating the impact of the intervention model and its implementation will provide the necessary tools to scale the intervention up to other CESFAMs across Chile.

**Clinical trial registration:**

[www.ClinicalTrials.gov], identifier [NCT05578066].

## Introduction

Chile presents one of the highest mental disorder burdens in the world, with nearly 38.3% of children and adolescents having had mental illness (1); one third of the population having had a psychiatric disorder in their lifetime, and 22.2% in the past year (2). Alcohol dependence accounts for 7.7% of total DALYs in Chile, and unipolar depression and anxiety disorders are at the top five of DALYs among women (3). The high prevalence of mental illness and substance use issues (MISUI) in Chile is confirmed by the results of the 2016–2017 National Health Survey (4). MISUI account for about 19% of global DALYs (5, 6) and in Chile, the longitudinal study “Mental Health Thermometer in Chile: Fifth Round” (2022) (7) concluded that 21.1% of participants suspected to have or had Mental Health Issue and 45.9% declared that they had a worse or much worse mood than before COVID-19 pandemic (7). There has also been an increase in the “sometime in life” consumption of non-prescription tranquilizers, hallucinogens, and pain relievers without a medical prescription in adults (8). The adolescent population is in first place in Latin American ranking of consumption of cocaine, marijuana, cocaine base paste and tranquilizers without a medical prescription (1, 5, 9).

Mental Health has been declared as a component of fundamental health human right, but in Latin America less than 40% of people with Mental Health disorders have received treatment (1). There is a global movement to strengthen and support Primary Health Care (PHC) services, including MISUI treatment (10, 11). The high prevalence of mental disorders among PHC patients and the fact that most patients with MISUI will access the health care system through PHC providers makes it an ideal setting to implement early screening and treatment strategies for these health problems (12). Evidence suggests that PHC may resolve up to 90% of mental health issues (13).

The gap in MISUI treatment represents a long-standing neglect of mental health care, with a variety of factors limiting access to care (14), including (1) PHC services lacking the ability to adequately respond to needs for MISUI treatment, and (2) stigma playing a significant role in the hesitancy of people with MISUI to recognize their condition or seek help (15, 16).

Stigma is a phenomenon comprising negative thoughts and actions toward the bearer, in which “elements of labeling, stereotyping, separating, status loss, and discrimination co-occur in a power situation that allows these processes to unfold” (17). Stigma affects multiple health domains such as social relationships, levels of stress, self-perception opportunities or behavior, and can add to the burden of disease or disability (18, 19).

Factors affecting stigma and discrimination are interacting constantly generating complex experiences of stigmatization (20, 21). In recent years, structural discrimination as well as socio-economic and political factors impacting stigmatized people has been described (21, 22). Professional stigma has also been described, as health professionals emulating socially stigmatized lay perceptions of those with mental illness (23).

Stigma toward people with MISUI is a global public health problem (21, 24, 25), and represents a main challenge in the integration of mental health into PHC (12, 26). People with MISUI are exposed to different stigma manifestations components that interact as an interrelated multilevel system, jeopardizing their mental health (20). MISUI stigma-related attitudes can be defined as the predisposition or tendency to respond that is triggered by a marker of illness (27). MISUI stigma-related behaviors refers to the discriminatory acts that result from the negative attitudes and stereotypes (28). Stigma-related attitudes and behaviors can be experienced in health care settings in various forms, such as being threatened with coercive treatment, being provided with insufficient information, being regarded as lacking the capacity for responsible action and being patronized or humiliated (21, 29).

It is important to recognize that there are differences in the way stigma manifests toward mental illness (MI) and substance use issues (SUI) (30–36). Literature suggests that people with SUI may have worse patient experiences when compared with patients without SUI (26). This represents an important challenge for accessing care for people with MISUI in PHC (37).

**FIGURE 1:**
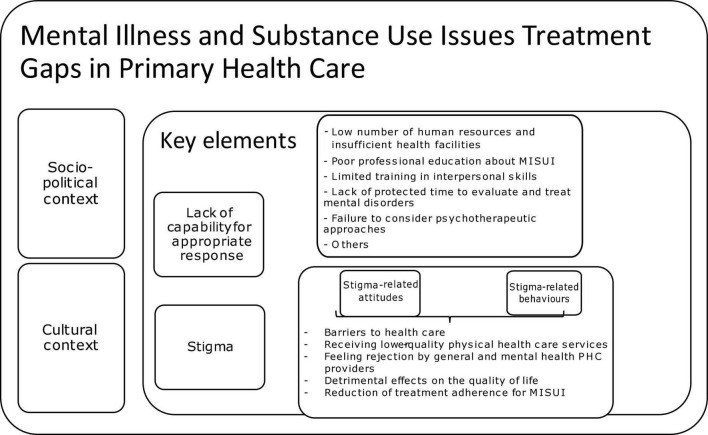
Gaps in MISUI treatment in primary health care.

Stigmatizing attitudes and actions from health professionals toward those with MISUI are barriers to health care (38, 39) and can lead to individuals with MISUI receiving lower-quality physical health care services than others (40, 41). Figure 1 describes MISUI treatment primary gaps in PHC, which interacts constantly with the sociopolitical and cultural context. There is ample evidence of MISUI stigmatization in health care, such as PHC settings (26, 42), and by health professionals (30–34). Recent (26, 42) studies have found that PHC physicians “don’t take mental illness as seriously as other chronic diseases” and negative attitudes toward people with substance use disorder are common among them (35, 36). These experiences of stigma can have detrimental effects on the quality of life of those receiving care (29, 43) and lead to a reduction of treatment adherence and outcomes, as well as perceived health for MISUI (44, 45). Stigma has also been identified as an important limitation in the help-seeking process (46) and mental health care access (23), also, perceived health care provider stigma may lead to worsened clinical and personal recovery (44, 47).

Services for MISUI at the PHC level have potential to minimize stigma and discrimination (23, 46), as people with these conditions are generally treated by the same providers and in the same community-based location as people with other health conditions (48).

There are other health professional-related characteristics limiting the access for optimal mental health care, including (i) poor professional education about MISUI, (ii) lack of training in interpersonal skills, (iii) inadequate time to evaluate and treat mental disorders, (iv) failure to consider psychotherapeutic approaches, among others (39).

**FIGURE 2:**
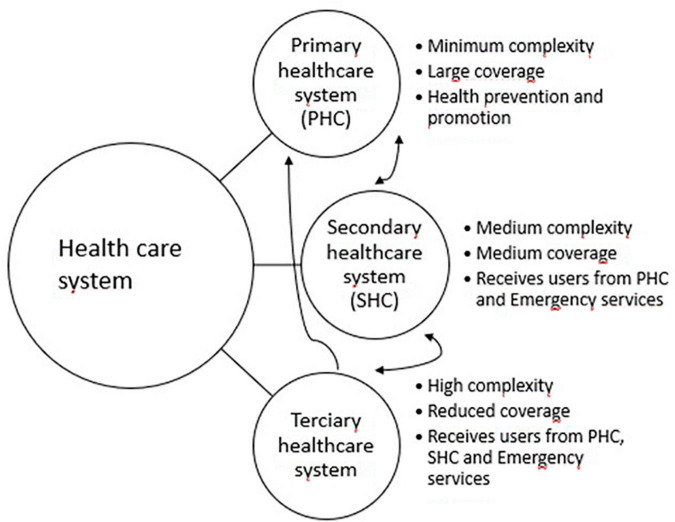
Chile’s public health care system.

The Chilean public health system (Figure 2) serves more than 75% of the population with its highest coverage in the middle- and low-income population groups (49, 50). Health service provision is organized by territory, through 29 Servicios de Salud (health districts) (15, 51). These health districts direct and coordinate activities from prevention to treatment and rehabilitation. They are organized through a hierarchical system that includes Tertiary and Secondary health care levels, as well as PHC (15, 52). Tertiary level has a reduced coverage and high complexity services. Secondary level has a medium coverage and complexity services. PHC provides services with high coverage, having diagnosis and treatment access available for most health problems, including MISUI. There are more than 500 PHC centers, in urban and rural areas, in which doctors, nurses, psychologists, social workers, technicians, and other providers work (53). Chilean PHC centers have implemented a Family and Community Health approach (54), becoming family health centers, or Centros de Salud Familiar (CESFAMs). Health professionals within the CESFAM are organized by territorial areas and provide care for the most common diseases. For MISUI, the CESFAM have the role of making a timely diagnosis, providing a set of treatments (based on clinical practice guidelines), or referring patients to a specialized mental health center (55).

Chile has been at the forefront of integration of mental health and PHC in Latin America (49, 52, 55–57). However, a significant gap for MISUI treatment exists, as only 38.5% of patients in Chile with a psychiatric diagnosis receive any kind of mental health care, whether treatment is provided by a specialist or by PHC (15). According to Chile’s National Mental Health Plan, up to 2017 Chile’s Mental Health budget was close to 2% of the overall Health budget, far below the 5% proposed by World Health Organization (3, 58). Although there has recently been an increase in national interest to enhance Mental Health budget.

The 2017–2025 National Mental Health Plan emphasizes enhancing the community mental health model, with a central role for PHC (3). Chilean PHC has already incorporated effective mental health programs, such as for depression or substance use problems, but there are challenges regarding low adherence levels (51, 53).

It is critical to test context-specific interventions to address stigma in Chilean PHC (2, 59, 60). The 2017–2025 National Mental Health Plan recognizes stigma toward MISUI as an important challenge (3). Evidence based-interventions that can effectively reduce MISUI stigma within health care settings are needed in Chile and worldwide (32, 61). However, a comprehensive, MISUI stigma reduction intervention is required and has not yet been tested or implemented in the Chilean PHC context. This is the first Chilean study to evaluate a comprehensive, multicomponent, anti-MISUI stigma intervention targeting CESFAM providers from an organizational perspective. There was another study specifically aimed at reducing stigma between PHC professional toward people with severe mental disorder diagnosis (62).

One of the projects to address stigma-related attitudes regarding MISUI in the Canadian PHC system was successfully implemented in three Toronto community health centers, resulting in the creation of a comprehensive anti-stigma intervention (21, 63, 64). The intervention proved to be effective at reducing stigmatizing attitudes among health professionals toward people with MISUI. Later on, that intervention was tested in Lima, Peru, after being adapted to the local context (21, 65).

Health care providers are an ideal target group for these interventions, given their clinical interactions with people with MISUI, however, stigma reduction programs for this group are uncommon (66), especially in PHC (61). Interventions comprising training specifically regarding stigma, social contact with users with MISUI, and a focus on recovery are most effective in terms of short-term improvements in stigma (67, 68). There is an important need to follow-up to determine whether positive intervention effects are sustained over time (66, 69). There is a need to actively include those with MISUI in the intervention process and to culturally adapt interventions carried out in settings other than those in which they were designed (70).

A previous related FONDECYT project (34) was just completed, a mixed methods study which sought to examine and understand the phenomenon of stigma toward people with MISUI in the PHC setting of the public health system in Chile. The study confirmed the presence of stigma toward people with MISUI. In addition, it (1) adapted and validated instruments to measure stigma among PHC providers in Chile (71), and (2) identified key elements to be considered for designing an effective intervention to reduce that stigma. In addition, it explored feasibility of the Canadian intervention. This research proposal represents a natural next step aimed at determining the effectiveness of a comprehensive anti-stigma intervention in reducing stigmatizing attitudes and behaviors among PHC providers toward individuals with MISUI in the Chilean context, using CESFAMs as the point of intervention.

## Methods and analysis

### Study design

**FIGURE 3:**
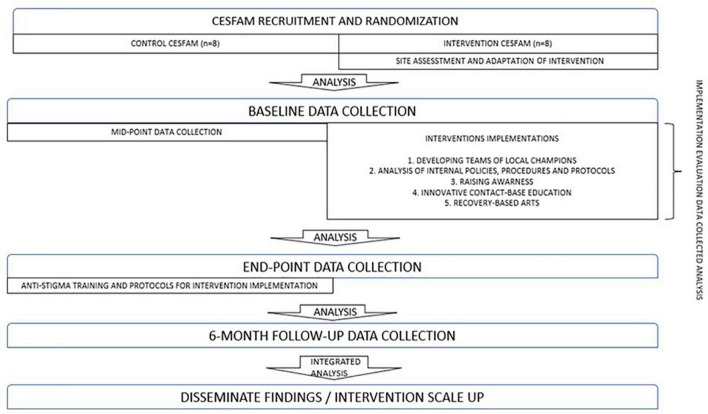
Study design.

This two-arm, cluster randomized controlled trial (64) will test the effectiveness of the adapted anti-stigma intervention for MISUI in Chile. As described in Figure 3, CESFAMs will be randomized to control and intervention arms of the study. Situational assessments will be conducted at intervention CESFAM, in order to understand their specific characteristics. A cultural adaptation will also be done at these PHC Centers, through a sequential process based on Barrera y Castro framework (72). Data will be collected in intervention and control CESFAMs on relevant stigma outcomes before (baseline), during (mid-point), and after (end-point) the intervention, as well as 6 months post-intervention (follow-up), to determine the effectiveness of the stigma reduction program. Additionally, data will be collected throughout to evaluate intervention implementation. The intervention stage will last a total of 18 months. This will be consist on five components that will be addressed furthermore. After developing the final integrated analysis, the team will disseminate findings and create a scale up intervention.

This research design is useful for non-clinical interventions that are targeted at health providers and patients, and has been used in PHC settings in the past (73, 74). Because an entire group (or “cluster”) is randomized to either intervention or control, the risk of contamination across trial groups is minimized (75). In the proposed study, the cluster will be the CESFAM, with eight intervention and eight control sites participating in the study. All PHC providers currently employed at the intervention CESFAMs and some PHC users that have received care there for MISUI in the 3 months prior to study participation are the intervention target groups. The intervention will be conducted in selected CESFAMs by the research team and/or dedicated and trained personnel. CONSORT guidelines for cluster randomized trials will be followed in all steps of the study (76).

### Inclusion and exclusion criteria

To be eligible for inclusion in the study, CESFAMs must serve a registered population of at least 15,000 people and have at least 50 staff employed. In addition, the following criteria will be considered: (1) geographic location; (2) characteristics of the population served (e.g., size, ethno-cultural profile); (3) rural or urban areas; (4) willingness to participate.

Exclusion criteria: Being part of another anti-stigma program.

### Sample, recruitment, and randomization of Centros de Salud Familiars

*Servicios de Salud* will be approached and invited to participate. CESFAMs that satisfy the inclusion/exclusion criteria within participating *Servicios de Salud* will be progressively invited to participate in the study. About 50% of CESFAMs in the Metropolitan Region will be recruited, 25% in the North and 25% in the South of Chile. Once the 16 CESFAMs that satisfy the criteria are selected, they will be randomly assigned to intervention and control conditions, within each of the three mentioned geographical areas. The nature of the intervention and cluster randomized design of the study requires application to the entire CESFAM (census approach), not to individual PHC providers.

### Intervention program

#### Study process

**FIGURE 4:**
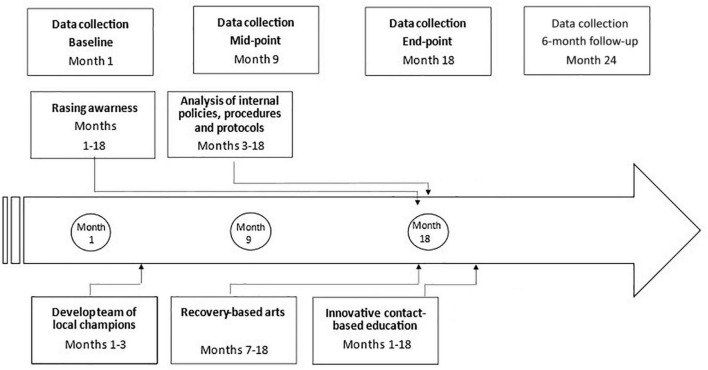
Intervention implementation process.

The overall implementation of the intervention process lasts 18 months, in which five main components are to be implemented: raising awareness about stigma and its effects on MISUI PHC users, developing a team of local champions, innovative contact-based education, analysis of internal policies, procedures, and protocols, and recovery-based arts. The entire stigma reduction intervention process will be developed between months 13 and 36 (Figure 4).

To exemplify the intervention program and timeline process, they will be described briefly accordingly to its specific objectives and their corresponding components.

#### Specific objective 1: Adapt the anti-stigma intervention with input of primary health care providers and users

An exploratory research and adaptation of the intervention will be developed during months 1–12, before the intervention. Once CESFAMs are recruited, situational assessments will be conducted in order to better understand the specific characteristics of each CESFAM to determine the logistics of implementing the intervention locally. Special emphasis will be placed on identifying the cultural and socio-demographics features of the organizations and the communities being served, to ensure that the intervention is tailored to address their specific needs. The assessment will include requests for the following information: (1) Overview of the CESFAM (history, mission, values, vision, strategic directions), (2) Organizational chart (full description of all services and programs, with an emphasis on MISUI), (3) Demographics of users, (4) Demographics of staff, (5) Challenges and opportunities, (6) Expectations about the intervention.

Based on the framework for cultural adaptation by Barrera and Castro (72), this study includes a sequential process to adapt the anti-stigma/pro-recovery intervention developed in the Canadian setting, the FONDECYT 1160099 project (34) results, and a Community-Based Participatory Research (CBPR) consultation process. CBPR in health is a collaborative approach to research (77), in which community and researchers abilities are acknowledged. CBPR involves the collaborative participation of researchers and the community that will be affected by research in the design and process of an intervention (78). It aims to combine knowledge and action for social change to improve community health and eliminate health disparities (77). This approach is implemented in order to improve the intervention relevance, adaptability and validity for the community. It also allows to access to PHC workers and users valuable information regarding internal processes and dispositions.

#### Specific objective 2: Implement a mental illness and/or substance use issues stigma reduction intervention in Centros de Salud Familiar

The comprehensive, 18-month, recovery-oriented anti-stigma intervention is composed of five components: (1) Developing a Team of Local Champions, (2) Analysis of Internal Policies, Procedures and Protocols, (3) Raising Awareness, (4) Innovative Contact-Based Education and (5) Recovery-based Arts. Teams of leaders developed as part of the first component will assist the research team with the implementation of the anti-stigma intervention at their respective CESFAM.

The first component, “Developing a team of local champions,” consists of 3–5 PHC providers and 1–2 users at each intervention CESFAM and it will be developed in months 1–3 of the intervention. The teams will comprise some individuals who have provided critical support and input, including participating in the exploratory research phase and the CBPR adaptation of the intervention framework for the Chilean context. The teams of local champions will assist with the data collection process throughout the study by encouraging colleagues to complete questionnaires and recruit users, as well as oversee and implement the intervention at their respective CESFAMs. These teams of champions will receive training to develop their skills as leaders and support the implementation process at their CESFAM. A self-administered evaluation questionnaire will be used to assess the effectiveness of the champion training and teams of local champions will track their activities at their own CESFAM.

Regarding the second component “*Analysis of Internal Policies, Procedures and Protocols*” it will be developed during months 3–18. This component involves evaluating CESFAM policies, procedures, and protocols using an anti-stigma/pro-recovery approach to identify strengths and areas for improvement in service delivery for individuals affected by MISUI. This evaluation will be completed using a policy analysis tool developed specifically for this intervention during the Canadian project; it is based on existing frameworks, such as the *Health Equity Impact Assessment Tool (HEIA)* (79) and the *QualityRights Toolkit* (80). HEIA is a tool that can be used to identify and address potential unintended health impacts (positive or negative) of a policy, program or initiative on specific population groups through five steps; scoping, potential impacts, mitigations, monitoring an dissemination (79). QualityRights Toolkit delivers information and tools for assessing and improving quality and human rights standards in mental health and social care facilities (80). At least five policies, procedures or protocols will be selected by the local champions and then analyzed by the research team. Recommendations concerning health equity, prevention of stigma and recovery-oriented practices promotion for individuals with MISUI will be developed and shared following analysis. It will be expected from each intervention CESFAM to implement at least one recommendation and make the necessary efforts to intend to educate PHC providers about the policy change in months 7–18 of the intervention. The impact of the implemented policy change will be analyzed at end-point data analysis and 6-month follow-up.

The third component *“Raising Awareness”* will be conducted during months 1–18. Various forms of media will be used to raise awareness about stigma related to MISUI among PHC providers and users. This component will be implemented throughout the intervention. Aligned with the premises of the contact-based educational element, local champions at intervention CESFAMs will determine the type of media they would like to use; this may include images, film, music, or a combination of media. This can be posters, web platforms, social media, among others. The research team will work with local champions to develop messaging to include in the media and assist with showcasing these pieces within each intervention CESFAM. This will also depend on the CESFAM’s particular resources and media choices. This component impact will be evaluated through its acceptability, adoption, appropriateness and coverage.

The fourth component “*Innovative Contact-Based Education”* will be conducted during months 1–18, trough educational workshops in intervention CESFAM. It will include anti-stigma and recovery principles, along with specific MISUI topics relevant to PHC providers. Topics will be determined by findings from the exploratory phase, current research, best practices, and the perspectives of local champions. Topics may include (1) supporting CESFAM PHC providers in preventing stigma and promoting recovery in their practice and (2) enhancing the competencies of CESFAM PHC providers for discussing MISUI with their users, identifying MISUI signs and symptoms, and referring users to psychosocial centers for treatment. Special emphasis will be placed on cultural beliefs and values that may influence stigma related to MISUI, concurrent disorders, and inter-professional collaboration within CESFAMs and between various health agencies.

The key feature of these workshops is the contact-based educational element (81), where people with lived experience participate in developing and delivering the curriculum to CESFAM PHC providers. mhGAP materials (82) will be used as main curriculum resources. As an incentive and recognition, a diploma will be given to participants. This component will be evaluated by the training team after each workshop to determine feasibility, coverage and perceived workshop usefulness.

Finally, the *“Recovery-Based Arts”* will be developed through months 7–18. Local champions at intervention CESFAMs will select one PHC provider member and recruit one artist to develop an arts curriculum and facilitate the art sessions. The facilitators will select an art form (e.g., painting, sculpting, music, sewing etc.) to use throughout the sessions. The facilitators, in collaboration with local champions, will determine themes related to MISUI to cover in each session. Ten users affected by MISUI and at least three CESFAM PHC providers will participate in the workshops each week. At the end of the 10-week program, each CESFAM will host an exhibition to showcase the artwork that has been produced.

#### Specific objective 3: Evaluate the effectiveness of the intervention in primary health care

This part of the interventions is composed by a quantitative and a qualitative component.

1. a Quantitative Component:

All PHC providers at the selected CESFAMs who have direct contact with users will be recruited for the study and will be expected to participate at all data collection time-points. A total sample size of at least 36 PHC providers per CESFAM with a total of 288 per arm its estimate (or 576 per data collection). Sample size for the proposed study was calculated in two steps. First, it was calculated with individual randomization. This calculation was based on the mean ± SD score for the OMS-HC scale was 48 ± 8.3 points, estimated with a sample of 798 PHC providers surveyed in FONDECYT N° 1160099 (34). This should be the average obtained in control CESFAM for the present study. Based on prior interventions in Canada (63), an estimated effect size of 10% in the intervention arm and a placebo effect of 3% in the control arm (from survey application) is expected. Thus, the average OMS-HC score in the intervention arm is expected to decrease to 43.2 ± 8.3 in the intervention group and 46.6 ± 8.3 in the control group (representing a decrease of 4.8 and 1.4 points, respectively). The standard deviation was assumed to remain the same in the intervention and calculations. To detect a statistically significant difference between intervention and control CESFAM, with an α = 5% and statistical power of 80%, the minimum sample size per arm is 94 individuals. Considering a 10% loss to follow-up in the intervention arm, 105 individuals per arm should be recruited with an individual randomization design. The minimum sample size was also calculated considering cluster randomized controlled design. Two elements were considered: (1) a minimum sample size of 105 and (2) an estimation of the intracluster correlation coefficient (ICC) for the study outcome (OMS-HC scale) (83). In this case, it was assumed the ICC to be ρ = 0.05, which is consistent with highest value reported in the literature for outcomes in primary care settings (74) and slightly higher than that reported in a study of a depression program in CESFAMs in Chile (84). Given the required sample size calculated based on individual randomization and the value of ICC = 0.05, the minimum number of clusters required is equal to: [105*0.05] = 5 clusters (75). However, a larger number of clusters would allow recruitment of a smaller number of individuals per cluster, maintaining an α = 5% and statistical power of 80% to detect differences between the control and intervention groups (85). For example, if 8 clusters per arm is considered (16 CESFAM total), it is necessary to recruit a minimum of 36 professionals per CESFAM. Thus, a minimum of the total sample of PHC providers to recruit would be 288 per arm (576 total). The validated, Chilean version of the Opening Minds Scale for Health Care Providers Scale (OMS-HC) (86), will be used as the primary outcome measure for PHC Professionals in the study. It will be applied at baseline, mid-point, end-point, with an expected 9-month interval between applications, and at 6-month follow-up.

A self-administered questionnaire will be used to examine stigma directed at persons with MISUI among health professionals. Five existing scales to measure stigma toward MISUI were selected to include in the questionnaire because they had been validated in Chile and were recommended by the research team. These scales reflect the current state of knowledge about stigma measurement (with a focus on health providers and persons with MISUI), as well as the feasibility of implementation at CESFAMs. They include: the Opening Minds Scale for Health Care Providers OMS-HC (83, 87, 88), Mental Illness: Clinicians’ Attitudes (MICA) (89), Modified Bogardus Social Distance Scale (90, 91) or Grandon Social Distance Scale (92), Recovery Scale for Providers (RS) (93), and the Recovery Self-Assessment-Revised (RSA-R) scale (94).

This questionnaire will be completed at four time-points (baseline, mid-point, end-point, and 6-month follow-up). It will collect data related to two main components: (1) socio-demographic and other relevant general variables; and (2) attitudes toward MISUI stigma and recovery. The end-point questionnaire will also include a third component focusing on the intervention and its implementation. A self-administered questionnaire will be provided to PHC providers at each CESFAM (intervention and control) at a time set aside by CESFAM directors.

CESFAM users will be recruited for participation in baseline and end-point data collection regarding their experiences of stigma by PHC providers. These users must be over the age of 18, have received treatment for MISUI at the CESFAM in the 3 months prior to participation (though not necessarily by a mental health professional), and have a MISUI diagnosis. User MISUI may be diagnosed by a health practitioner or self-diagnosed. Since stigma is a barrier to seeking and receiving help, it is important to include participants who have not been formally diagnosed by a healthcare provider. A research team member will approach users at the waiting room and screen them for inclusion and exclusion criteria. Users in capacity to consent will be invited to sign the consent form and those who sign will be part of the face-to-face survey assisted by a research team member on the same day in the same health facility. To assess capacity to consent, a Spanish adaptation of dimensions and criteria of the Macarthur Competency Assessment Tool for Clinical Research (MACCAT-CR) (95) are used. This process includes the following actions; (1) The interviewer exposes the user to the relevant information of the project after which he/she is questioned about what has been explained (comprehension); (2) Subsequently, they are asked about their appreciation or assessment of the information provided in their specific circumstances; and (3) A reasoned reflection on the decision to be made is promoted, assessing the circumstances and consequences, to end up expressing their choice.

The primary outcome for users will be measured using The Internalized Stigma of Mental Illness (ISMI) scale, validated for the Chilean population (96). The study will collect cross-sectional samples at baseline and end-point data collection and compare average scores between intervention and control groups for baseline and end-point measures.

A minimum sample size of 27 PHC users per CESFAM was calculated (or 216 users per study arm, or 432 users total per data collection). For this sample size calculation, the mean ± SD for the ISMI score was 10.34 ± 4.74 points, estimated based on the results of FONDECYT N° 1160099 (34). A placebo effect of 1% was considered in the control group, and a 15% expected effect size was considered in the intervention group, based on the protocol for a similar intervention study completed in Canada (which estimated a higher effect of 19.5%; a more conservative estimate was selected for this calculation) (64). Thus, the mean of the scale would decrease to 10.23 ± 4.74 in the control group and to 8.79 ± 4.74 in the intervention group (an average decrease of 0.1 and 1.55 points, respectively). No change in the standard deviation was assumed, to be conservative with the calculation. To detect a statistically significant difference of this size between intervention and control groups, with a significance level of α = 5% and statistical power of 80%, 169 individuals are required in each group (intervention and control). Loss to follow-up it is not considered, as individuals recruited at the baseline and end-point data collection will not necessarily be the same, and this will be a cross-sectional sample. In the cluster RCT design, it is expected that the ICC of the ISMI score would be relatively low, as the CESFAM user population is more likely to be heterogeneous than the PHC providers. Assuming an ICC = 0.01, and considering the k = 8 clusters in each arm of the study (from PHC provider calculations), or 16 CESFAM total, it is necessary to recruit a minimum of 27 PHC users per CESFAM, or 216 users in each arm, for a total sample size of 432.

A face-to-face survey assisted by a research team member will be used to examine how users perceive stigmatizing attitudes and behaviors among CESFAM PHC (97) providers. The questionnaire will include four main components: (1) socio-demographic and other relevant general variables; (2) perceived stigmatizing attitudes and behaviors among CESFAM PHC providers; (3) perceived recovery-oriented practices by CESFAM PHC providers; and (4) accessing healthcare at their CESFAM. Subjective experience of stigma as conveyed by CESFAM PHC providers will be measured among patients using validated tools. The Perceived Devaluation-Discrimination Scale will be used to assess the extent to which users believe that other people devalue or discriminate against someone with MISUI. This scale has shown acceptable internal consistency (*a* = 0.78) (97). The tool is validated in Chile (98). An adapted version of the Discrimination Experience Subscale of the 29-item ISMI scale was designed to measure the subjective experience of stigma, e.g., respondents’ perceptions of how they are treated by others. It measures alienation, stereotype endorsement, perceived discrimination, social withdrawal, and stigma resistance. The Cronbach’s alpha value of the total score was 0.83 and the Spearman-Brown Coefficient of 0.76 (99). For FONDECYT N° 1160099 (34), a shortened version of the ISMI scale was used, which was validated for use in Chile with an (*a* = 0.916) (96). The Person in Recovery Version of the RSA will also be considered to assess users’ perceptions of recovery-oriented practices in their CESFAMs. Due to the sensitive nature of the study scales, there is some risk of social desirability bias in participants’ responses. Both PHC providers and users will complete the Marlowe-Crowne Social Desirability Scale (MCSDS). The MCSDS will allow to measure and control such bias. The scale has 33 items which were defined to be culturally acceptable but unlikely to occur, and to have minimal abnormal implications for either the socially desirable or socially undesirable responses (100).

1. b Qualitative component:

To evaluate implementation of the intervention, qualitative interviews will be held with two PHC providers (local champions) and one CESFAM authority per intervention site at baseline, mid-point, and end-point. In addition, at least one local champion from the community will be interviewed to explore his/her/they experience as a champion, pros and cons of the intervention and how it could be improved. Questions will relate to implementation outcomes for the intervention: acceptability, adoption, appropriateness, feasibility, fidelity, implementation cost, coverage and sustainability (101). In addition, mid-point, end-point, and follow-up questionnaires administered for PHC professionals in experimental CESFAMs will include questions regarding: acceptability, adoption, appropriateness, feasibility, fidelity, implementation cost, coverage, and sustainability. End-point interviews will also include questions regarding key elements needed for scaling up the intervention. In addition, near to the culmination of the project, a Symposium with 20–30 key stakeholders including members from the Ministry of Health, *Servicios de Salud* and CESFAMs, among others, will be held in order to define final recommendations to scale up the anti-stigma intervention on a national level. The discussion process will be guided through ExpandNet & WHO Framework (102) and the recent theoretical recommendations from Greenhalgh and Papoutsi (103) regarding scaling up processes in health, as well as the critical aspects about dissemination of stigma reduction interventions identified by Kemp et al. (69).

*Specific Objective 4* corresponding to “*identify critical barriers and opportunities for its implementation in PHC”* and *Specific Objective 5, “Develop recommendations to scale up the anti-stigma intervention”* will be developed by the qualitative component of data previously mentioned.

**FIGURE 5:**
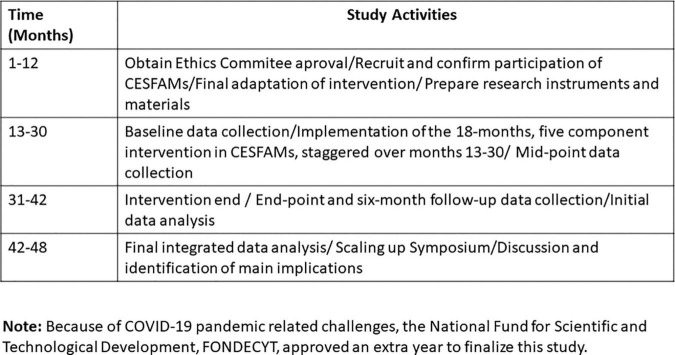
Study activities.

A brief summary of the study’s activities is described, correlated with the project timeline in Figure 5.

This intervention is expected to promote the following outcomes; Participation in the experimental group will result in a significant decrease in stigmatizing attitudes among PHC providers toward individuals with MISUI compared with the control group as measured by the Chilean version of the OMS-HC; Participation in the experimental group will result in a significant decrease of PHC users experiences of stigma conveyed by PHC providers compared with the control group as measured by the ISMI scale, validated for the Chilean population; The changes in attitudes and behaviors within the experimental group will be sustained over time as measured at 6 months-follow-up.

### Data analysis

The data analysis will be conducted through months 42–48. Post data collection activities consider the following: (1) Data cleaning and evaluation, (2) Creation of derived variables, (3) Response rate calculation and (4) Bias evaluation.

It will be carried out using SAS 9.4 (specifically, using POC MIXED and PROC GENMOD) and R 3.5. Mixed-effect modeling will used for both PHC provider and user data, which will include individuals and CESFAMs as random effects and group (intervention/control) as fixed effects. The technique is appropriate to analyze cluster randomized controlled trials because these models can account for the possible dependence between responses of users and PHC providers within the same CESFAM. A descriptive analysis will initially be conducted to obtain a general picture of the sample. Time and important covariates (e.g., demographics) as they relate to our outcome variable will be explored. Univariate association tests will be performed to clarify the unconditional effect of these covariates on outcomes.

After the quantitative and qualitative data have been analyzed, summarized, and interpreted independently, the primary focus of the integrated analysis will be on identifying and discussing to what extend and in what ways the qualitative results help to explain the quantitative results (explanatory design). Implementing a “hybrid” approach (104) that will be used for qualitative data.

## Discussion

This study represents a new stage of relevant and innovative research in mental health and stigma in Chile that will contribute to improving access and quality of care for people with MISUI. Evaluating the impact of the intervention model and its implementation will provide the necessary basement to scale the intervention up to other CESFAMs across Chile. This intervention is vital to fight stigma toward MISUI and other conditions in PHC and the Chilean health system overall. Knowledge translation will be a special focus of this study, in order to communicate results to local, national, and international audiences. It is also important to evaluate the feasibility of the intervention scale up, since different studies have reported cultural influences on mental illness-related stigma (105).

Stigma has a detrimental effect on health policies (106), treatment outcomes (107), and efficient and effective recovery from mental health problems (23). Evidence indicates that stigma reduction initiatives must be comprehensive, multifaceted, and able to target various levels within a setting. Different strategies to address stigma have been suggested (66, 69). At the organizational level, specific interventions implemented across entire institutions (e.g., workplaces) (108) may provide supportive environments that encourage anti-stigmatizing practices (109). It has been suggested that reducing stigma interventions with people already in contact with health services, as people with MISUI, needs alternative strategies to deal with self-stigma and cope with experienced stigma to facilitate adherence (46). That reinforces the importance of a collaborative approach, where service users and healthcare practitioners work toward destigmatize PHC. Healthcare professionals stigmatizing behaviors and beliefs may be subtle and denied because of how they are perceived (23) thus, it is important to implement interventions focusing on awareness, internal policies, procedures and protocols. It’s also relevant to implement educational approaches, as the one included in this protocol, as adequate information and contact between the public and the stigmatized individuals would lead to diminish stigma (110) in PHC settings.

This study has some particular limitations and potential bias: (a) Non-response bias: A survey of this kind will invariably tend to select the more cooperative and communicative respondents, who may also be more tolerant. Different strategies have been considered to increase the response rate, even among people who might have more stigmatizing attitudes. In particular, it is important to consider potential item non-response as a limitation of this study. The research team will emphasize respondents the importance of trying to answer all the questions and some alternatives, like mean substitution or other imputation methods, will be used if is necessary; (b) Social desirability bias: As with other measurement approaches, there are potential biases measuring stigma attitudes, because it might be considered a sensitive issue. Self-administered questionnaires expect to reduce this bias, as well as the respect for confidentiality and use of the MCSDS (100); (c) Difficulties to measure attitudes: It can be difficult translating untouchable concepts into variables; (d) External validity is threatened by the limitations of the sample, the generalizability of the results are limited to the target population (CESFAM PHC Providers); (e) Inferring behavioral responses from reported intentions; (f) Non-blinding: Participants will not be blinded. Since the intervention is an RCT, this may be a bias source.

Some of the main strengths of this study include: (a) There is a real public health need for this intervention study: stigma is a key factor that affects people with MISUI, resulting in their reluctance to seek health care services. This study will be one of the first to intervene to reduce stigma among PHC professionals in Chile. As discussed in Background, prior stigma reduction intervention work has found that reducing MISUI stigma in PHC has the potential to increase access to care for user with these conditions, improve their quality of life, and contribute to improved treatment adherence for MISUI. (b) The use of a census/organizational approach: seeks to change organizational culture and stigma toward those with MISUI *via* contact-based education, structural policy change, and raising awareness at the CESFAM level. (c) Cultural appropriateness: Special efforts will be made to adapt the intervention for this context, and the stigma instruments were adapted to the Chilean context in FONDECYT 1160099. The mixed methods approach of this research allows addressing many of the limitations of the quantitative stigma measures and facilitates deep understanding of intervention’s impact and implementation. (d) The inclusion of both health providers and users in this study: Many studies of stigma in PHC have not included the perspective of users, limiting the impact of their results in the lives of people with MISUI.

After a critical analysis of the proposed study, it is possible to say that its design and internal validity are sufficiently strong and that special measures have been taken to control and reduce its potential limitations. Finally, it is important to remark that “stigma” has many sources and this study will not be able to tacked all of them (e.g., media, social services, the educational system, and legislation). While recognizing stigma as a complex concept, this study seeks to reduce stigma at the health services level by an innovative and collaborative approach. Having that in mind, this will be a unique relevant study to test an innovative anti-MISUI stigma intervention targeting CESFAM providers in Chile from an organizational perspective.

## Ethics and dissemination

a. Research Ethics Approval

This protocol and the template and site-specific informed consent forms, recruitment materials and other requested documents were reviewed, analyzed and approved by the sponsor and the applicable Pontificia Universidad Católica de Chile Ethics Committee (EC) (ID::190603010), Herminda Martin de Chillán Hospital EC, Valparaiso Health Service EC, Coquimbo Health Service EC and Reloncavi Health Service with respect to scientific content and compliance with applicable and intervention research and human subjects regulations. The proposal will follow all ethical guidelines provided for conducting research with human beings. The proposal, interim reports and final reports will be submitted to the EC’s at the beginning, middle, and end of the study.

b. Consent

All participants will be required to read and sign an informed consent form outlining the aims and objectives of the study prior to engaging in any aspects of the project, particularly before participating in the data collection process. It will be presented comprehensibly, the opportunity to ask questions will be given, understanding confirmation will be solicited, and voluntarily participation will be re assured. All user participants will also receive an oral explanation of the consent process prior to signing and/or agreeing to participate, and PHC user capacity to consent will be considered.

Informed consent will be conducted by interventors/researchers/PHC workers, guaranteeing adequate training and experience, in order to protect participants moral wellbeing and human rights. The consent process will be conducted by trained professionals.

c. Confidentiality

All participants will be assigned a numerical code, resulting in the anonymization of data. All knowledge translation materials will only include data for groups with 10 members or more to protect confidentiality. Although the quantitative surveys include a question about the participant’s CESFAM, reports for each CESFAM will be general and will not include a separate analysis by profession, limiting the potential to identify individual respondents.

d. Ancillary and Post-Trial Care

This study involves minor risk of potential harm (physical, emotional and/or social), however, specific measures will be taken to minimize them: (1) confidentiality, so participants will not be treated differently than other PHC providers/CESFAM as a result of their responses; (2) participation is voluntary and no negative consequences will result for those who decide not to participate; (3) participants may skip any/all questions they do not want to answer as part of the mixed methods approach; (4) information is provided to all participants regarding the institutions conducting the research, the principal investigators, and contact information and active referral for psychosocial support when needed for those who may be emotionally triggered by participating; and (5) final results will be shared with participants.

e. Dissemination Policy

The research team conceptualizes dissemination as a key component of a comprehensive knowledge translation approach (111) in a dynamic ongoing cycle. Special efforts will be made in all the stages of the project to make research results accessible to various audiences (such as research participants, *Servicios de Salud* and CESFAM authorities, Chilean Ministry of Health, and the academic community) through resources such as a research portfolio, reports, at least three academic papers, meetings, and at least two academic conference presentation, as well as to explore possibilities like articles and features in local mass media (e.g., radio, television, and newspapers). There will be a specific budget that supports dissemination efforts. Each academic or research report, will be reviewed by project research committees and by peers, prior to submission to evaluate methodology and implementation and appropriateness merits. The study results will be released to the participating PHC workers and users.

## Ethics statement

This study was reviewed and approved by the Pontificia Universidad Católica de Chile Ethics Committee, Herminda Martín de Chillán Hospital Ethics Committee, Valparaíso Health Service Ethics Committee, Coquimbo Health Service Ethics Committee, and Reloncaví Health Service Ethics Committee. The patients/participants provided their written informed consent to participate in this study.

## Author contributions

JS was the leading researcher. JS and PV conceived the study with the support of other research team members. AK, SB, PC, and JS participated in the design and implementation of a previous initial study in Canada. IB, JS, AK, and SB were part of an earlier similar study in Peru. JS, PV, TA, RA, MA, FP, LV, PB, CÁ-H, AJ, NV-M, SB, IB, AK, PC, and CT were part of the design and/or initial implementation of the protocol in Chile. FP, JS, and LV provided statistical expertise in clinical trial design. LV conducted the primary statistical analysis in the FONDECYT 1160099 study. All authors contributed to refinement of the study protocol and approved the final manuscript.
